# The Effect of Tear Supplementation on Ocular Surface Sensations during the Interblink Interval in Patients with Dry Eye

**DOI:** 10.1371/journal.pone.0135629

**Published:** 2015-08-24

**Authors:** Lóránt Dienes, Huba J. Kiss, Kristóf Perényi, Zsuzsanna Szepessy, Zoltán Z. Nagy, Árpád Barsi, M. Carmen Acosta, Juana Gallar, Illés Kovács

**Affiliations:** 1 Department of Ophthalmology, Semmelweis University, Budapest, Hungary; 2 Department of Photogrammetry and Geoinformatics, Budapest University of Technology and Economics, Budapest, Hungary; 3 Instituto de Neurociencias, Universidad Miguel Hernandez-CSIC, San Juan de Alicante, Spain; University of Illinois at Chicago, UNITED STATES

## Abstract

**Purpose:**

To investigate the characteristics of ocular surface sensations and corneal sensitivity during the interblink interval before and after tear supplementation in dry eye patients.

**Methods:**

Twenty subjects (41.88±14.37 years) with dry eye symptoms were included in the dry eye group. Fourteen subjects (39.13±11.27 years) without any clinical signs and/or symptoms of dry eye were included in the control group. Tear film dynamics was assessed by non-invasive tear film breakup time (NI-BUT) in parallel with continuous recordings of ocular sensations during forced blinking. Corneal sensitivity to selective stimulation of corneal mechano-, cold and chemical receptors was assessed using a gas esthesiometer. All the measurements were made before and 5 min after saline and hydroxypropyl-guar (HP-guar) drops.

**Results:**

In dry eye patients the intensity of irritation increased rapidly after the last blink during forced blinking, while in controls there was no alteration in the intensity during the first 10 sec followed by an exponential increase. Irritation scores were significantly higher in dry eye patients throughout the entire interblink interval compared to controls (p<0.004). NI-BUT significantly increased after HP-guar (p = 0.003) but not after saline drops (p = 0.14). In both groups, either after saline or HP-guar the shape of symptom intensity curves remained the same with significantly lower irritation scores (p<0.004), however after HP-guar the decrease was significantly more pronounced (p<0.004). Corneal sensitivity to selective mechanical, cold and chemical stimulation decreased significantly in both groups after HP-guar (p<0.05), but not after saline drops (p>0.05).

**Conclusion:**

Ocular surface irritation responses due to tear film drying are considerably increased in dry eye patients compared to normal subjects. Although tear supplementation improves the protective tear film layer, and thus reduce unpleasant sensory responses, the rapid rise in discomfort is still maintained and might be responsible for the remaining complaints of dry eye patients despite the treatment.

## Introduction

Dry eye with symptoms of ocular discomfort is one of the most commonly reported conditions in eye care with an estimated prevalence ranges from approximately 5% to 35% [1]. Patients who suffer from dry eye have varying levels of symptoms, such as ocular dryness, burning, photophobia, foreign body sensation and redness, and may or may not have clinical signs, such as rapid tear film breakup time, increased osmolarity, and increased ocular surface staining [2]. The cornea has rich innervation of different types of sensory receptors and selective stimulation of these nerve endings evokes distinct sensations [3]. The precorneal tear film protects the ocular surface from external damage but corneal nerve endings are exceedingly close to the surface and can easily react to different types of environmental stimuli, especially when the tear film is abnormal. Current opinion about the origin of the unpleasant sensations that accompany dry eye is that they are primarily due to abnormal activity of cold receptors secondary to ocular surface desiccation and tear hyperosmolarity [4,5]. 10–15% of the corneal nerve fibers are cold sensitive and they appear to be primarily involved in detecting external temperature variations, including those associated with tear evaporation [3]. Although the evaporation rate of the tear film is determined by multiple factors, including protein and aqueous components of the tear film and the mucin coating of the epithelial cells, the status of the lipid layer is crucial in the prevention of evaporation [6]. In particular, the thickness of the lipid layer affects evaporation and a thicker tear film lipid layer found to be correlated with better tear film stability and less symptoms of dry eye [6–8]. In healthy subjects ocular surface desiccation proved to be correlated with increasing ocular discomfort, suggesting that both tear film thinning and tear film breakup stimulate underlying corneal nerves, although tear film breakup produced more rapid stimulation of corneal sensory nerves [9].

Current therapy of dry eye is based on tear supplementation to lubricate, replace missing tear constituents and to decrease elevated tear film osmolarity and evaporation, thus reducing symptoms of irritation [8]. Long term use of artificial tears in patients with dry eye has been consistently associated with significant increase in tear film breakup time [10–14], and improvement in patient assessed parameters such as ocular comfort and dry eye symptoms [15–18]. However, patients seek not only for alleviation of complaints after prolonged tear supplementation but also for fast relief for dry eye symptoms. Although the decrease of ocular surface irritation after long term tear supplementation have already been described as a result of retarding precorneal tear film breakup, neither the characteristics of ocular surface sensations nor the short-term effect of tear supplementation on the unpleasant feelings in dry eye patients are clear. The evaluation of ocular surface sensations associated with tear film drying may help to understand the pathophysiologic changes of corneal sensory nerves that occur in dry eye and might be responsible for the complaints despite frequent tear supplementation. The aim of this study was to investigate the characteristics of ocular surface sensations and corneal sensitivity during the interblink interval in dry eye patients before and shortly after tear supplementation.

## Materials and Methods

Patients who had been diagnosed as having dry eye symptoms for at least 3 months, with an OSDI score of ≥13 evaluated by the questionnaire of Ocular Surface Disease Index (OSDI) [19] have been recruited for this study. Subjects who showed significant corneal staining (>Grade 2, Oxford Scale) [20] were excluded because corneal epitheliopathy could potentially be a confounding factor affecting ocular surface sensitivity [21–23]. Subjects with ophthalmic conditions other than dry eye or systemic disease including blepharitis, meibomitis, lid abnormalities as well as contact lens wearers were also excluded. None of the subjects received any drops at least 6 hours before the measurements. Participants in the control group did not have any clinical signs and/or symptoms of dry eye (OSDI score <10) or significant ocular surface disease and were not using eye drops.

First, blinking was tracked for 1 minute while the participant was reading a logMAR chart. The participant was unaware of the investigator counting the blinks. The interblink interval subsequently was calculated by dividing 60 seconds by the amount of blinks in that period. Tear film dynamics was assessed by non-invasive tear film breakup time (NI-BUT) in parallel with continuous recordings of ocular sensations during forced blinking. Corneal sensitivity to selective stimulation of corneal mechanonociceptors, cold receptors and chemical nociceptors were assessed using the Belmonte gas esthesiometer. Blinking rate, interblink ocular surface sensations and corneal sensitivity to the different stimuli were explored before and 5 minutes after instillation of a drop of sterile saline or hydroxypropyl-guar gellable lubricant eye drop (Systane Balance Lubricant Eye Drops; Alcon, Alcon Inc, Fort Worth, TX, USA) in one eye of each participants. During the experiments, the right eye was used for data collection and the left eye was closed with a patch. In order to prevent the long term effect of HP-guar on tear film dynamics, in all cases sterile saline was applied first, followed by the application of HP-guar drops 60 min later. All experiments were performed during the morning hours by the same physician with evaluating blinking rate at first, immediately followed by the assessment of NI-BUT and ocular surface sensations, and then by measuring corneal sensitivity. All procedures were repeated after the application of saline and HP-guar drops.

The study was conducted in compliance with the Declaration of Helsinki, applicable national and local requirements regarding the ethics committee and institutional review boards. Ethical approval was obtained from the Institutional Review Board (Semmelweis University Regional and Institutional Committee of Sciences and Research Ethics). A written informed consent was obtained before the examination from each patient.

### Measuring non-invasive tear film breakup time during forced blinking

The non-invasive tear film breakup time (NI-BUT) was measured using the Keeler Tearscope Plus (Keeler, Windsor, UK) immediately after a complete blink. The Tearscope Plus was attached to a Topcon SL-D2 slit lamp in a fixed position to obtain a full coverage of the cornea. The measurement of the non-invasive tear film breakup time with Tearscope Plus is based on the projection of a cylindrical source of cool white fluorescent light onto the cornea so that tear film breakup could be observed at any point over the corneal surface. The tear film was recorded by a Topcon DV-3 digital camera attached to the slit lamp, captured videos were exported at a spatial resolution of 1024 × 768 pixels and were analyzed by a masked observer. The non-invasive tear film breakup time was defined as the time from the last blink when visible deterioration of the projected rings was detectable during the continuous recording. In each subject, NI-BUT was averaged from three consecutive measurements.

### Recording symptom intensity during forced blinking

A specific instrument with a rotary potentiometer was built to record intensity rating continuously during forced blinking. Participants were instructed to adjust the potentiometer to the corresponding intensity of sensations arising during tear film drying, as it was described previously [9]. Briefly, subjects were trained before data collection to use the rotary potentiometer while viewing the current position of the potentiometer. After training, participants were instructed to continuously rate the sensation intensity with the potentiometer forcing the eye to remain open. Full counterclockwise rotation represented “no sensation” and rotating full turn clockwise represented “so intense that I must blink immediately”, similarly to a previous description [9]. Subjects were asked to focus on performing the sensation intensity ratings to minimize the lag between the sensations they experienced and their response with the potentiometer [9]. A specific computer software written in MatLab program (The MathWorks, Natick, MA) was used to sample the data acquired from the potentiometer and to convert it to numeric values on a 10 unit scale. Only individual full sets of scaling data were used. During data collection, the subjects received no feedback other than the kinesthesis from the potentiometer position. The data were collected continuously at 0.2-second intervals and was averaged from three consecutive measurements.

### Corneal sensitivity to suprathreshold selective stimuli

We measured with the potentiometer the irritation sensation evoked by selective mechanical, chemical, and thermal stimuli applied on the central cornea of participants using the original Belmonte gas esthesiometer. Suprathreshold intense mechanical (260 ml/min), chemical (75% CO_2_ in air), and cold (-3.9°C corneal temperature change) stimuli were used during three-second air pulses of adjustable flow rate, composition (CO_2_%) and temperature. First, mechanical threshold levels were determined by using methods as described previously elsewhere [24] with variable flows of medicinal air. Then, suprathreshold mechanical stimulus was applied at a constant flow of 260 ml/min. Air was heated at the tip of the probe at 50°C so that it reached the ocular surface at 34°C to prevent a change in corneal temperature caused by the airflow [25]. Thermal stimulation was done by cooling the air to produce the required changes in basal corneal temperature with a 10 ml/min flow below mechanical threshold. For chemical stimulation, a mixture of medicinal air with a concentration of 75% CO_2_ was used at 50°C at the tip of the probe and with a flow rate of 10 ml/min below mechanical threshold. According to the recommendations, medicinal air and medicinal CO_2_ were used to prevent any contamination of the ocular surface or the esthesiometer during the experiments. The measurements were made before and 5 min after tear supplementation and irritation responses were recorded with the potentiometer as described previously. Immediately after each gas pulse, the subject scored other sensation parameters: degree of irritation, stinging and burning pain, and warming and cooling components of the sensation.

### Statistical analysis

Statistical analysis was performed with SPSS software (version 21.0, IBM Corp. Armonk, NY). Sample size was based on a power calculation (power 0.90; p = 0.05) using SDs obtained in the previous studies from our institution. The Shapiro-Wilk W test was used to assess normal distribution of the variables and p values of less than 0.05 indicated that the data tested do not follow a normal distribution. Due to non-normality of data the Mann–Whitney U test was used for group comparisons and the Wilcoxon signed-rank test was applied to compare repeated measurements on a single subject using a Bonferroni correction for repeated measurements. In case of group comparisons a p value less than 0.05 was considered as statistically significant, in case of repeated measurements the Bonferroni adjusted p was set to 0.004.

## Results

Twenty eyes of twenty subjects (12 men, 8 women) were included in the dry eye group with a mean age of 41.88±14.37 years. Fourteen eyes of fourteen subjects (9 men, 5 women) with a mean age of 39.13±11.27 years were included in the control group. There was no statistically significant difference in age or gender distribution between the two study groups. The mean OSDI score was 30.19±15.49 in the dry eye group and 3.45±2.95 in the control group (p<0.001). The non-invasive tear film breakup time in the dry eye group was 4.16±2.44 sec, and in the control group was 13.05±6.01 sec (p<0.001).

### Ocular surface sensations during forced blinking

**Fig 1:**
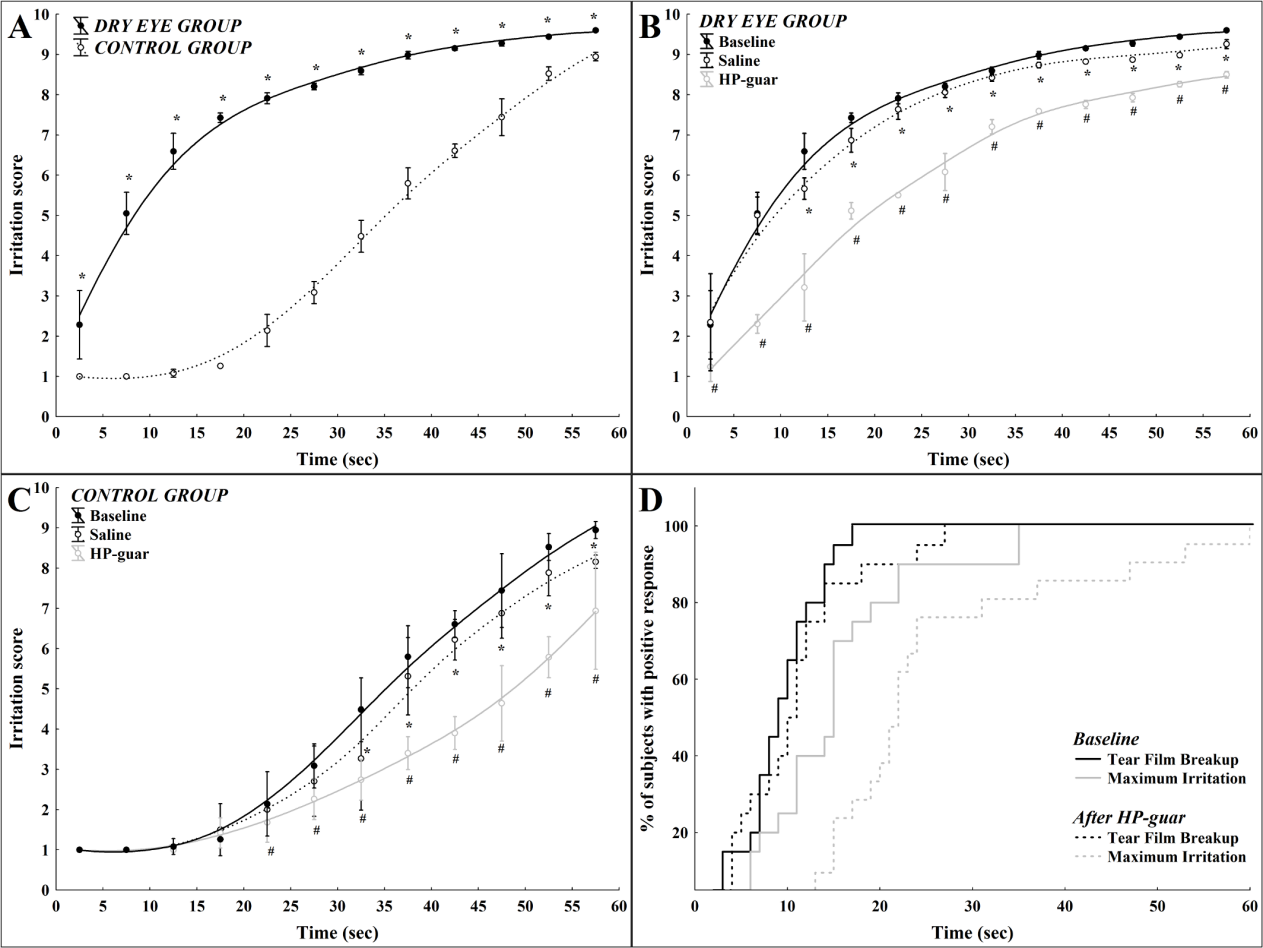
Mean irritation scores as a function of time during forced blinking in dry eye and in normal subjects. (**A**) Mean values of ocular irritation during the interblink interval in the control and in the dry eye group (*: between-group difference p<0.004) (**B**) Mean values of ocular irritation scores during the interblink interval after application of saline or HP-guar drops in the control group (*: baseline vs. saline p<0.004; ^#^: baseline vs. HP-guar p<0.004) (**C**) Mean values of ocular irritation scores during the interblink interval after application of saline or HP-guar drops in the dry eye group (*: baseline vs. saline p<0.004; ^#^: baseline vs. HP-guar p<0.004) (**D**) Cumulative distribution of NI-BUT and maximum irritation before and after application of HP-guar from both groups. Note: whisker: pooled variance for 5 sec time frames.

In dry eye patients the intensity of irritation quickly increased during forced blinking, the relationship between time and ocular surface irritation could best be described by a quadratic regression model during the interblink interval with an r value of 0.94 (r^2^ = 0.89, p<0.001, Fig 1A). On the contrary, sensation intensity followed a different curve in the control group: in the first 10 sec with no alteration in the intensity followed by an exponential increase of irritation score with time (r = 0.97, r^2^ = 0.94, p<0.001, Fig 1A). Sensory responses were significantly higher in the dry eye group compared to the normal group at every 5 sec timeframes during the interblink interval (p<0.004 after Bonferroni correction, Fig 1A).

### The effect of tear supplementation on ocular surface sensations during forced blinking

In the dry eye group, saline eye drop significantly reduced sensory responses after the first 10 sec during the interblink interval (p<0.004, Fig 1B). The application of a HP-guar lubricant drop also resulted in a significant reduction of the sensory responses throughout the entire interblink interval (p<0.004, Fig 1B). The difference between sensory responses after saline or HP-guar lubricant drops was statistically significant at every 5 sec timeframes during the interblink interval (p<0.004, Fig 1B).

In the control group, saline eye drop significantly reduced the sensory responses only after the first 30 sec during the interblink interval (p<0.004, Fig 1C). The application of a HP-guar lubricant drop resulted in a significant reduction of sensory responses after the first 20 sec during the interblink interval (p<0.004, Fig 1C). The difference between sensory responses after saline or HP-guar lubricant drop proved to be statistically significant at every 5 sec timeframes after the first 20 sec during the interblink interval (p<0.004, Fig 1C).

Tear supplementation with a HP-guar lubricant drop significantly increased the time to develop tear film breakup and maximum irritation during forced blinking (p = 0.01; Fig 1D). The time to intense (score ≥8) irritation responses significantly correlated with the values of NI-BUT both before and after tear supplementation with HP-guar in dry eye patients (before: r = 0.48, p = 0.03; after r = 0.62, p = 0.007) and also in control subjects (before: r = 0.55, p = 0.04; after r = 0.59, p = 0.01).

### The effect of tear supplementation on NI-BUT

Analysing the whole cohort, the average non-invasive tear film breakup time (8.18±3.28 sec) increased significantly after application of a HP-guar lubricant drop (10.44±4.44 sec; p = 0.003) but increased only a slightly after application of saline (9.86±4.96 sec; p = 0.14). The average increase in NI-BUT was 31% after application of HP-guar and 17% after application of saline drops independently of the initial NI-BUT value. In the dry eye group, the interblink interval increased from 3.77±2.59 sec to 4.11±2.13 sec (p = 0.48) after application of saline and to 5.52±2.84 sec (p = 0.01) after application of HP-guar. In the control group, the interblink interval increased from 6.23±2.21 sec to 7.19±2.55 sec (p = 0.11) after application of saline and to 8.13±3.03 sec (p = 0.01) after application of HP-guar.

### Effect of tear supplementation on corneal sensitivity

Sensations evoked by mechanical stimuli were defined by all subjects as irritating with a predominantly stinging component. The sensation evoked by CO_2_ was defined by all subjects as irritating, with stinging, burning, and pricking components. Significantly lower irritation was reported after cold stimulation, with mild irritating, sometimes cooling components.

**Fig 2:**
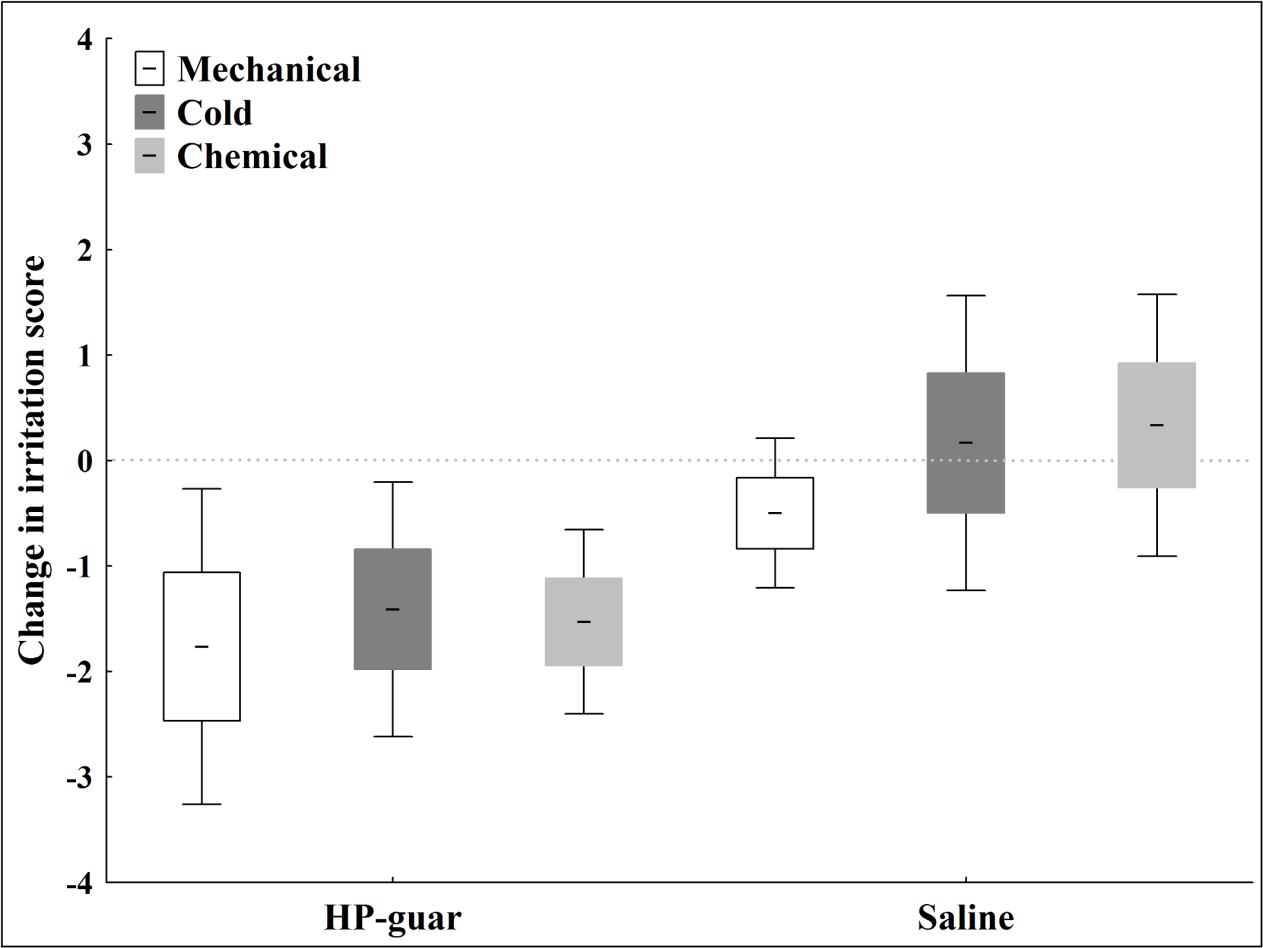
Relative decrease in irritation scores to selective stimulation of corneal nerves after tear supplementation in the dry eye group. The change in irritation scores to mechanical, chemical and cold stimulation after application of saline and HP-guar drops. Note: line: mean; box: standard error of mean; whisker: 95% confidence interval of mean.

Although decreased, the application of saline drop had no significant effect on corneal sensitivity to mechanical, cold or chemical stimulation evaluated by the gas esthesiometer in either group (Table 1). However, five minutes after application of HP-guar drop, corneal sensitivity to mechanical, cold and chemical stimulation decreased significantly in both groups (Table 1). Fig 2 demonstrates the mean decrease in irritation scores to selective mechanical, cold and chemical stimulation after tear supplementation in the dry eye group.

**Table 1:** Change of irritation scores to selective stimulation of corneal nerves after tear supplementation.

	Baseline	After saline		After HP-guar	
	*Mean ± SD*	*Mean ± SD*	*P*	*Mean ± SD*	*P*
**Dry eye group**					
***Mechanical***	4.9 ± 2.9	4.4 ± 1.5	0.23	3.1 ± 2.9	0.001
***Chemical***	7.1 ± 1.1	6.8 ± 2.7	0.68	5.6 ± 1.7	0.006
***Cold***	2.1 ± 2.3	1.9 ± 3.0	0.81	0.9 ± 2.4	0.04
**Control group**					
***Mechanical***	6.3 ± 2.0	5.6 ± 1.9	0.12	4.3 ± 2.2	0.001
***Chemical***	7.0 ± 2.4	6.5 ± 2.0	0.28	5.6 ± 2.0	0.006
***Cold***	2.0 ± 1.6	1.7 ± 1.9	0.49	1.0 ± 1.9	0.02

Mean ± SD values of irritation scores to mechanical, chemical and cold stimulation at baseline and after application of saline or HP-guar drops. Note: P compared to baseline, Wilcoxon signed-rank test.

## Discussion

Dry eye syndrome is a common cause of ocular irritation and the primary management goals are to restore the natural homeostasis of the ocular surface and tear film and thus improve the patient’s ocular comfort and quality of life [26–28]. There are several reports with documented improvement of subjective symptoms and some objective parameters (tear film stability, ocular surface staining) after tear supplementation in the long term [14–18,27], but in most cases frequent administration of eye drops is required to maintain symptom remission. Although appropriate treatment resulting in prompt relief of complaints is the desirable medium of care, the relationship between ocular surface drying and the onset of unpleasant sensations has not yet been clarified in this population.

In this study we demonstrated, that in dry eye patients the characteristics of ocular surface sensations during the interblink interval is considerably different from that in normal subjects. In healthy control subjects we found a delayed increase in sensation intensity between two complete blinks, with a three-phase curve: first a brief period (< 10 sec) with no alteration in the intensity followed by a relatively flat and then, by a steeper slope similarly to the results of a previous study evaluating healthy subjects [9]. In healthy control subjects, the relationship between time and ocular surface sensations could be best described with an exponential curve in contrast to dry eye patients, where a sudden increase of sensations was found after the last blink. Here we describe for the first time, that in dry eye patients ocular surface irritation increases rapidly after the last blink and is significantly more pronounced throughout the entire interblink interval compared to healthy subjects. Previously it has been speculated, that in healthy subjects, the sensory level is returned to a “basal” state during blinking and remains at this level during minimal or no tear film alteration [9]. It is possible that the early onset of unpleasant sensations observed in dry eye patients might be the result of an inability to establish this symptom-free basal state during blinking. The clinical relevance of this finding is that not only tear film dynamics but the characteristics of ocular surface symptoms are substantially different in dry eye patients, which should be taken into account when their relationship is evaluated. Although dry eye patients had decreased tear film breakup time compared to controls, lower NI-BUT did not result in a simple leftward shift of the characteristic symptom intensity curve of normal subjects, but we observed a substantially different intensity curve suggesting altered excitability of corneal receptors in this population. After tear supplementation with either saline or HP-guar lubricant drops, symptom intensity curves followed the same pattern with significantly lower irritation scores suggesting that normalized tear film leads to a better protection of the cornea but does not have a direct effect on the altered ocular surface sensations.

The assumption of improved protection of ocular surface by the normalized tear film layer is also supported by our results on corneal sensitivity measured with the Belmonte noncontact esthesiometer. In this study, when analyzed ocular surface irritation as a response to selective stimulation of corneal receptors, five minutes after the application of HP-guar lubricant eye drop corneal sensitivity to mechanical, cold and chemical stimuli were significantly lower compared to corresponding values before tear supplementation. Although decreased, the application of saline drop had no significant effect on corneal sensitivity in either group. The mean decrease in corneal sensitivity after application of HP-guar was similar for cold, mechanical and chemical sensations suggesting that normalized tear film decrease the final intensity of the stimulus reaching the corneal nerve endings. Previously it has been proposed, that in the case of stimuli with cold air, evaporation is the main contributing factor to the cooling effect and the magnitude of evaporation primarily depends on tear film stability [29] which was substantially better after tear supplementation with HP-guar as indicated by significantly increased tear film breakup time. In the case of mechanical stimuli, the tear film is expected to act as a limited filter for mechanical forces and an increase in its thickness and/or elastoviscosity would decrease the transmission of force to the nerve endings so that the same stimulus would be less intensely felt. This effect has already been demonstrated after intra-articular injections of hyaluronan solutions where the reducing action on joint nociceptor discharges appeared to depend predominantly on their role as an elastoviscous filter associated with their rheological properties [30]. Chemical stimulation with CO_2_ is conceivably mediated by the protons, resulting in the local formation of carbonic acid in the microenvironment of the nerve endings. Carbonic acid formation is known to be proportional to CO_2_ concentration which is less affected by the thickness of the tear film [31].

Studying tear film dynamics may provide a better understanding of mechanisms involved in subjective symptoms of dry eye, however dynamic measurement of the sensations as a result of ocular surface drying is problematic. Instruments such as verbal and computer-based category scales are especially useful for continuous psychophysical rating of pain as the patient’s report is considered to be the most valid measure of subjective experiences such as discomfort and pain [32]. To quantify ocular surface irritation during forced blinking we built a digital potentiometer to enable the simultaneous collection of continuous sensory and tear film dynamics data. To assess tear film dynamics during forced blinking we measured the non-invasive tear film breakup time. Tear film dynamics has long been studied using different methods including laser interferometry and tear film breakup time, and results have shown that the tear film continuously changes during the blinking cycle [33–38]. When analyzed over a long time, a gradual decrease in central tear film thickness could be observed [39,40], and a close correlation between tear film breakup and sensation was described [9,33]. It has also been reported, that after tear supplementation precorneal tear film thickness increases up to 40 micrometers and remains significantly elevated for at least 5 minutes [38,40,41]. However, to the best of our knowledge, there is no available clinical data on the effect of improved tear film stability on ocular surface sensations in the short term. In this study we tested the effect of tear supplementation using sterile saline or a HP-guar gellable lubricant eye drop containing micro-emulsions of oils. HP-guar gellable lubricant eye drops contains propylene glycol, hydroxypropylguar, borate, and sorbitol, and additionally includes both a polar phospholipid surfactant and mineral oil to mimic the lipid layer of the tears and thus to minimize the evaporative loss of tears from the ocular surface. According to previous reports, significant improvements in lipid layer thickness and tear film breakup time were seen after HP-guar eye drop, and these beneficial effects lasted at least for 60 minutes [42,43]. In our study, five minutes after one drop of HP-guar eye drop we observed a significant increase in non-invasive tear film breakup time with a concomitant decrease in ocular sensations during forced blinking or selective stimulation of corneal sensory nerves. Compared with HP-guar, saline eye drops only marginally improved tear film stability, and the decrease in ocular surface sensations was significantly less pronounced suggesting that in the short term the direct protective effect of the normalized tear film might be responsible for the decreased corneal sensations. This assumption is also supported by other findings of this study, such as the significant correlation between the tear film breakup time and the onset of intense irritation responses both before and after tear supplementation, and the significant increase in interblink interval shortly after the application of HP-guar drops.

The finding that corneal sensitivity against cold, mechanical and chemical stimulus significantly decreased after tear supplementation helps to explain the clinical observation, that there is a rapid beneficial effect of improved tear film stability on patient complaints. It is known, that corneal cold receptors which is responsible for maintaining basal tear secretion [44] are able to discriminate transient temperature variations of 0.5°C, such as tear film evaporation. The selective stimulation of corneal cold receptors in humans by less than 3°C decrease in temperature evokes distinct, conscious sensations of cooling that become increasingly unpleasant when larger temperature decrease is applied. It has also been demonstrated, that stimulation of a significant part of the population of polymodal nociceptor fibers and/or of mechanonociceptor fibers results in unpleasant feeling and reflex tearing [45].

The Belmonte non-contact esthesiometer allows exploration of different types of sensory fibers, such as mechanosensory fibers that respond to mechanical forces; polymodal nociceptive fibers that respond to mechanical forces, irritants, extreme temperatures, and endogenous inflammatory mediators; and cold fibers that are activated mainly by the decrease of temperature [24,25]. The good reproducibility of mechanical, heat and chemical threshold measurements using noncontact esthesiometers has been previously reported [31,46–48]. According to a study in healthy volunteers, one drop of sodium hyaluronate reduced the irritative components of the sensation evoked by selective stimulation without affecting the ability of subjects to identify the intensity of the stimuli [49] allowing us to make direct comparisons of ocular surface sensitivity before and after tear supplementation.

Although we have demonstrated a relationship between ocular sensations and tear film dynamics before and after tear supplementation, changes that occur during tear film disruption and their relation to the sensations induced are not well understood. Tear film hyperosmolarity, alteration in the lipid layer and transmembrane mucin gel layer, deterioration of the hydrophilic mucous of the ocular surface have been postulated as common events during tear film breakup [50]. The increasing intensity of ocular sensations resulting from dryness could be the result of these alterations [51,52]. In this study, there was no significant difference in corneal sensitivity to mechanical, cold and chemical stimulation between the dry eye and the control groups demonstrating that the sensory function of corneal neurons remained virtually intact in this population. However, taken together these findings with the results on ocular surface sensations, it is reasonable to conclude, that alterations in the excitability of corneal receptors can precede the development of corneal hyposensitivity in dry eye patients. Our results strongly suggest, that the pathophysiology of symptoms in patients with dry eye are considerably different from normal subjects and further studies are recommended as this difference would be better described when longitudinal data of patients with the entire spectrum of the disease were analyzed. Our future analyses aim to examine whether tear supplementation leads to the decrease of symptoms primarily as a result of better ocular surface protection or whether the improvement in ocular surface alterations (corneal epitheliopathy and corneal nerve damage) is the main reason for clinical improvement in the long term.

One might conclude from our results that the decrease in ocular surface sensations shortly after tear supplementation might be the consequence of a thicker and more stable tear film which decrease the intensity of environmental stimuli reaching the ocular surface. In our opinion, the fact that tear supplementation with HP-guar decreased ocular surface sensations, blinking frequency and sensitivity to selective stimulations supports the assumption that improved tear film dynamics has an important role in protecting the ocular surface, and thus in decreasing unpleasant feelings. Several previous studies have aimed to describe the ocular surface retention time and the effect on tear film thickness of the different artificial tear formulations. Mean ocular surface residence time of 23.5 min and a 30% increase in tear film thickness was reported after one drop of 0.3% sodium hyaluronate using quantitative gamma scintigraphy [53]. These results are compatible with data obtained using optical coherence tomography that show that 0.15% sodium hyaluronate eye drop increase the tear film thickness by 20% for 40 minutes [54]. On the contrary, after application of one drop of polymer-free 0.9% sodium chloride, neither significant increase in tear film thickness nor retention time of >20 min were demonstrated [53,54] which might explain the modest effect of saline drop on ocular surface protection in our study. Moreover, the short ocular surface retention time of saline allowed us to assume the lack of significant effect of saline drop on tear film dynamics 60 min after its application in our experimental protocol.

As a conclusion, in this study we have shown, that not only tear film dynamics but the characteristics of ocular surface sensations are also substantially different in dry eye patients compared to normal subjects. We have also shown, that improved tear film dynamics has a beneficial effect on ocular surface protection against environmental stimuli but has no direct effect on the altered characteristics of sensations. Based on these results we may conclude that in spite of the rapid improvement in tear film dynamics after tear supplementation, altered ocular surface sensations are still maintained and might be responsible, at least in part, for the remaining complaints reported by numerous dry eye patients despite treatment.
